# Supplementary material for: Sugar transporter Slc37a2 regulates bone metabolism in mice via a tubular lysosomal network in osteoclasts
Source: Nat Commun. 2023 Feb 21;14:906. doi: 10.1038/s41467-023-36484-2 (PMC9945426; doi:10.1038/s41467-023-36484-2)
Supplement: Supplementary file 1 — Supplementary Information [file 41467_2023_36484_MOESM1_ESM.pdf]

SUPPLEMENTARY INFORMATION

Sugar transporter Slc37a2 regulates bone metabolism in mice via a tubular lysosomal network in osteoclasts

Ng, Ribet, Guo et al.

Supplementary Table 1. Details of commercial antibodies used in the Methods section.
Dilutions used for immunofluorescence (IF) and immunoblotting (IB) are indicated.

Antibody	Clone	Supplier	Catalogue #	Dilution
**Primary Antibodies**				
Mouse monoclonal anti-Actin	JLA20, deposited by Lin, J.J.-C	DSHB	JLA20	IB 1:2000
Rabbit monoclonal anti-Akt (pan)	C67E7	Cell Signaling Technology	4691	IB 1:1000
Rabbit monoclonal anti-Phospho-Akt (Ser473)	D9E XP®,	Cell Signaling Technology	4060	IB 1:1000
Mouse monoclonal anti-ARL8A/B	H-8	Santa Cruz	sc-398635	IF 1:200
Mouse monoclonal anti-Cathepsin K	182-12G5	Millipore	MAB3324	IF 1:300
Mouse monoclonal anti-Cathepsin K	E-7	Santa Cruz	sc-48353	IB 1:250
Rabbit polyclonal anti-Cathepsin B		Atlas Antibodies	HPA018156	IB 1:250
Mouse monoclonal anti-CD63/LAMP3	H5C6	DSHB	H5C6	IF 1:100
Rabbit polyclonal anti-Collagen I		Abcam	ab34710	IF 1:300
Rabbit polyclonal anti t-ERK1/2		Promega	V1141	IB 1:1000
Mouse monoclonal anti-p-ERK1/2	E-4	Santa Cruz	sc-7383	IB 1:500
Mouse monoclonal anti-GM130	35/GM130	BD Transduction Laboratories	610822	IF 1:100
Rabbit polyclonal anti-IκBα	C-21	Santa Cruz	sc-371	IB 1:1000
Rat monoclonal anti-LAMP-2	ABL-93, deposited by August, J.T	DSHB	ABL-93	IB 1:1000
Rat monoclonal anti-LAMP-2	GL2A7, deposited by Granger, B. L	DSHB	GL2A7	IF 1:100
Mouse monoclonal anti-NFATc1	7A6, deposited by Crabtree, G.R	DSHB	7A6	IB 1:1000
Rabbit polyclonal anti-MMP13	ab39012	Abcam	ab39012	IF 1:300
Mouse monoclonal anti-PDI	1D3	ENZO Life Sciences	ADI-SPA-891-F	IF 1:200
Rabbit polyclonal anti-Rab1b		Santa Cruz	sc-599	IB 1:5000
Mouse monoclonal anti-Rab5	621.3	Synaptic Systems	108 011	IB 1:1000
Rabbit monoclonal anti-Rab7	D95F2	Cell Signaling Technology	9367	IF 1:100
Mouse monoclonal anti-Rab7	E9O7E	Cell Signaling Technology	95746	IB 1:2000
Mouse monoclonal anti-Rab38	A-8	Santa Cruz	sc-390176	IB 1:200
Rabbit monoclonal anti-RUNX2	D1L7F	Cell Signaling Technology	12556	IF 1:200
Rabbit polyclonal anti-Syntaxin 16		Synaptic Systems	110 163	IB 1:300
Mouse monoclonal anti-c-Src	GD11	Millipore	05-184	IB 1:1000
Mouse monoclonal V-ATPase D1	34-Z	Santa Cruz	sc-81887	IB 1:1000
Rabbit polyclonal anti-VDAC3	H-40	Santa Cruz	sc-292328	IB 1:500
Rabbit polyclonal anti-VGluT-1		Synaptic Systems	135 303	IB 1:1000
Mouse monoclonal anti-Vps35	B-5	Santa Cruz	sc-374372	IF 1:300
**Secondary Antibodies**				
Goat anti-mouse IgG (Fab specific)-Peroxidase antibody		Sigma-Aldrich	A9917	IB 1:5000
Goat anti-rabbit IgG (whole molecule)-Peroxidase antibody		Sigma-Aldrich	A0545	IB 1:5000
Alexa Fluor 488 goat anti-mouse IgG (H+L), highly cross-adsorbed		Thermo Fisher Scientific	A-11029	IF 1:500
Alexa Fluor 568 goat anti-mouse IgG (H+L), highly cross-adsorbed		Thermo Fisher Scientific	A-11031	IF 1:500
Alexa Fluor 647 goat anti-mouse IgG (H+L), highly cross-adsorbed		Thermo Fisher Scientific	A-21236	IF 1:500
Alexa Fluor 488 goat anti-rabbit IgG (H+L), highly cross-adsorbed		Thermo Fisher Scientific	A-11034	IF 1:500
Alexa Fluor 568 goat anti-rabbit IgG (H+L), highly cross-adsorbed		Thermo Fisher Scientific	A-11036	IF 1:500
Alexa Fluor 647 goat anti-rabbit IgG (H+L), highly cross-adsorbed		Thermo Fisher Scientific	A-21245	IF 1:500
Alexa Fluor 555 donkey anti-rat, IgG (H+L), highly cross-adsorbed		Thermo Fisher Scientific	A-48270	IF 1:500

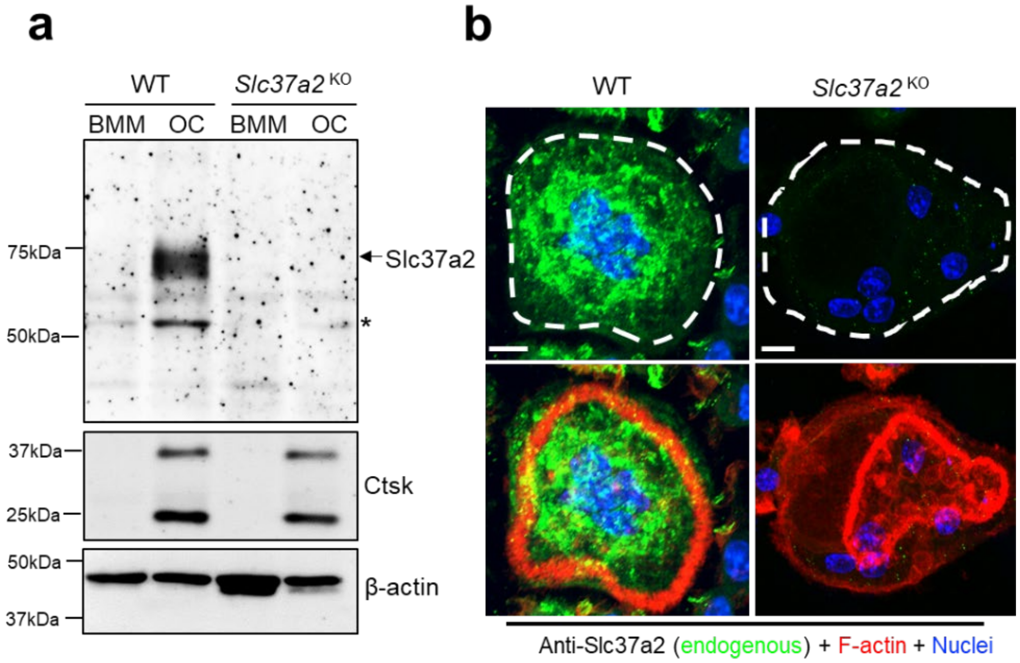

Supplementary Figure 1. **Specificity of custom-made antibody against Slc37a2. a** Western blot preparations of WT and *Slc37a2* ^{KO} bone marrow monocytes (BMM) and osteoclasts (OC) to confirm for specificity of a custom-made rabbit anti-Slc37a2 antibody directed against the cytoplasmic facing intracellular loop (against the peptide sequence CTPPRHHDDPEKEQ). A major band corresponding to the predicted size of glycosylated Slc37a2 is indicated by the black arrow and a minor species of unglycosylated Slc37a2 is indicated by the asterisk (*). Little to no Slc37a2 signal was detected in WT BMMs. The signal was lost in *Slc37a2* ^{KO} derived BMMs and OCs. Cathespin K (Ctsk) was used as a marker of OC differentiation and β-actin served as a loading control. 30µg of post-nuclear lysate were loaded per lane for each sample (n = 3). Source unprocessed blots are available in Source data. (b) Immunofluorescence staining of WT and *Slc37a2* ^{KO} OCs to validate the specificity of the Slc37a2 antibody (n = 3). Strong Slc37a2 signal (green) is detectable within rhodamide-phalloidin labelled F-actin rings (red) of WT bone-resorbing osteoclasts but is absent within F-actin rings of *Slc37a2* ^{KO} OCs. Nuclei (blue) were visualized with Hoechst 33342. Scale bar: 10 µm.

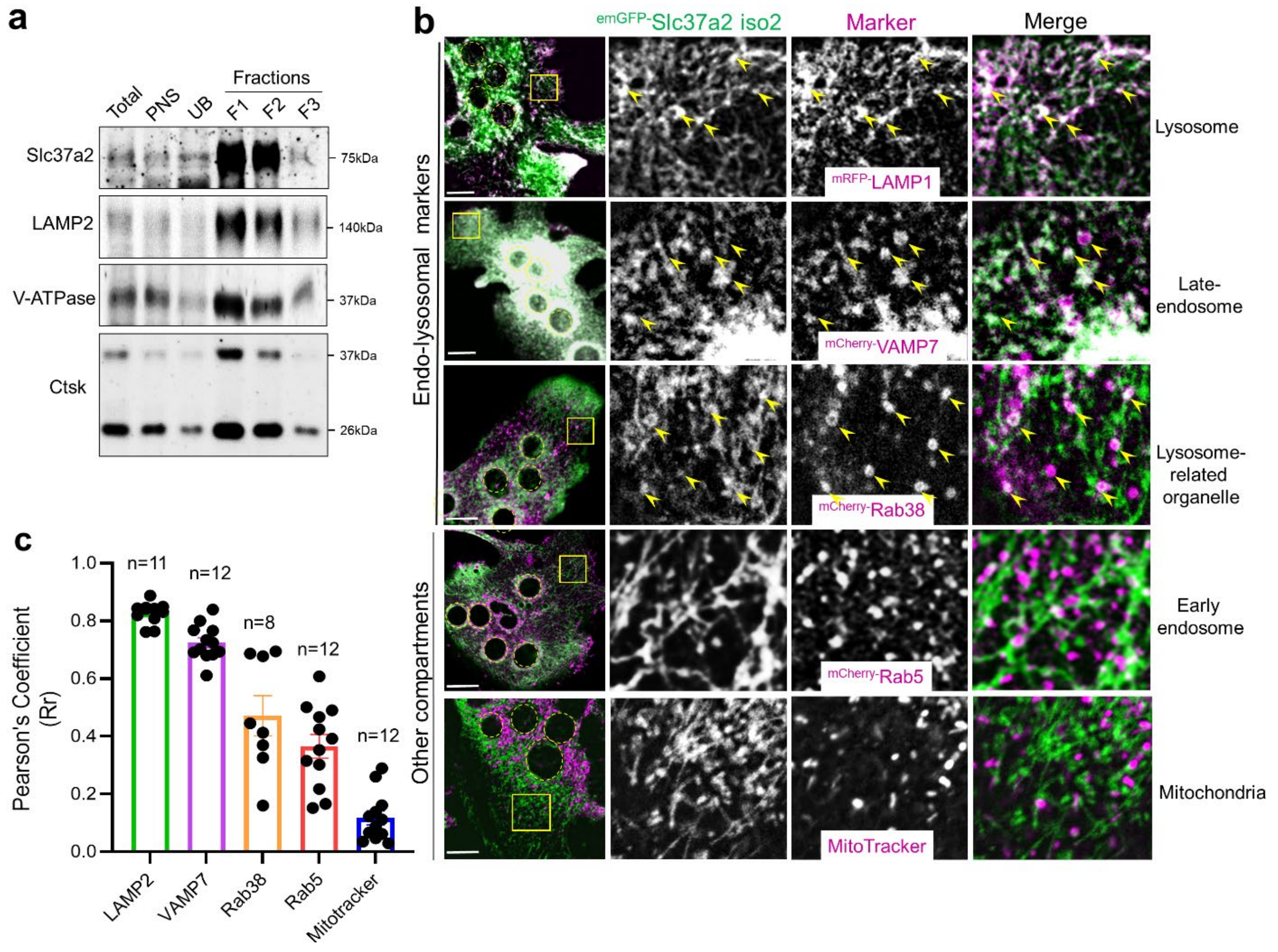

Supplementary Figure 2. Slc37a2$^+$ tubular SLs colocalize with endolysosomal markers in living osteoclasts, Related to Fig. 3. (a) Slc37a2 co-enriches with SLs. 5 µg of proteins of indicated fractions collected during the isolation of enriched SLs was analyzed by SDS-PAGE and immunoblotting for Slc37a2 and SL markers LAMP2, V-ATPase and Ctsk. **(b)** Representative confocal images of live BMM-derived mouse osteoclasts on glass expressing $^{emGFP\text{-}}$Slc37a2 isoform 2 together with indicated organelle markers. Arrows denote co-labeled organelles in magnified enlargements. Bar, 10 µm. (**c**) Colocalization analyses as determined by Pearson's correlation coefficient (Rr) (*n*= 8-12 cells per group). Mean ± SD. Source data are provided as a Source Data file. See also Supplementary Movie 1 & 2.

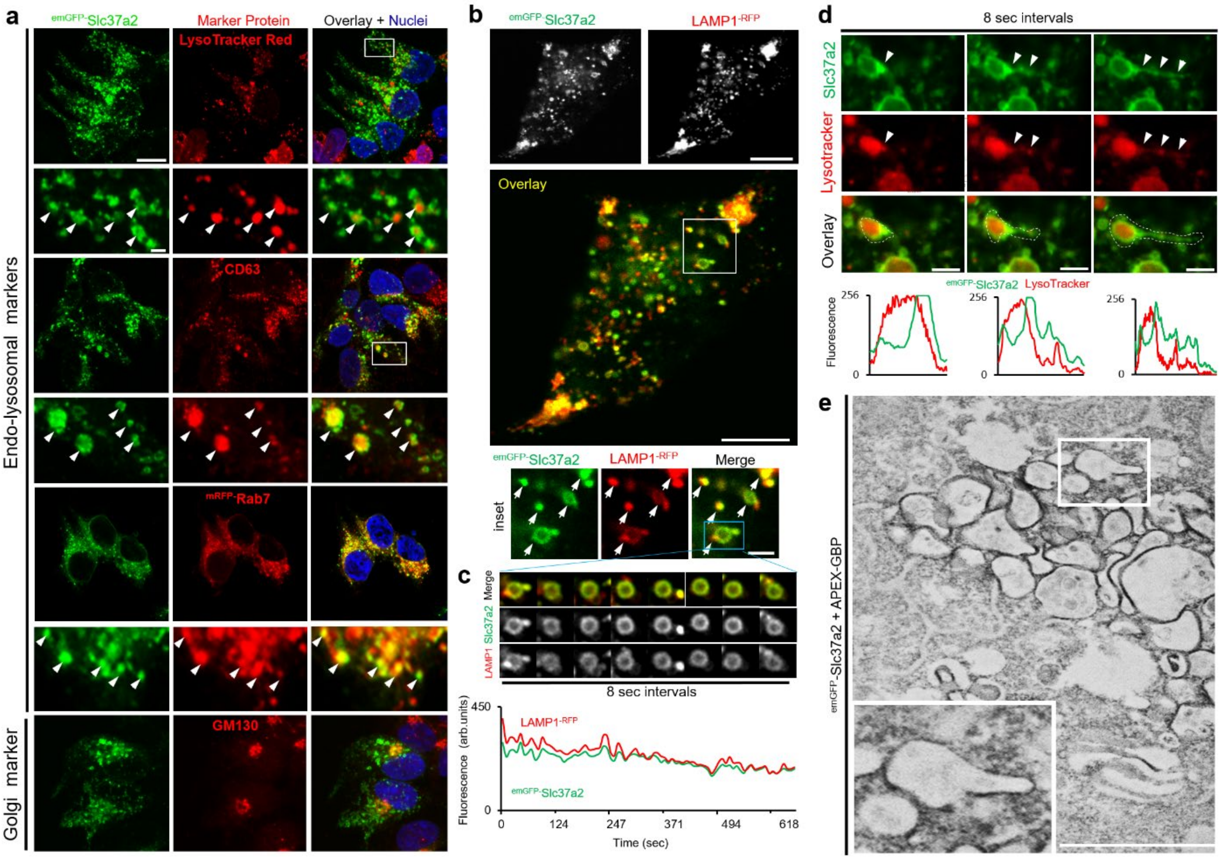

Supplemental Figure 3. emGFP-Slc37a2 localizes to late-endosomes/lysosomes in non-osteoclastic cell lines. Related to Fig. 3. (**a**) Representative confocal images of HEK293 cells expressing emGFP-Slc37a2 with indicated organelle markers. Bar, 10 μm (n = 3). (**b-c**) Live cell confocal imaging of a HEK293 cell co-expressing emGFP-Slc37a2 with LAMP1-RFP (Bars, 10 μm). Magnified region highlights colocalization of puncta (Bar, 1 μm) and blue boxed region corresponds with time-lapse series shown for an individual endolysosome shown in (**c**) with associated intensity signal of fluorophores shown over time, intervals = 8 sec, (n = 2). (**d**) High-resolution time-lapse series of an emGFP-Slc37a2 labeled endolysosome co-labeled with Lysotracker Red. Corresponding line scans are shown below, intervals = 8 sec. Bar, 1 μm (n = 3). (**e**) Detection of emGFP-Slc37a2 using a co-expressed APEX-tagged GFP-binding protein (APEX-GBP) in BHK cells results in prominent DAB reaction product labeling of late endosomes and lysosomes. Bar, 1 μm (n = 2). Source data are provided as a Source Data file.

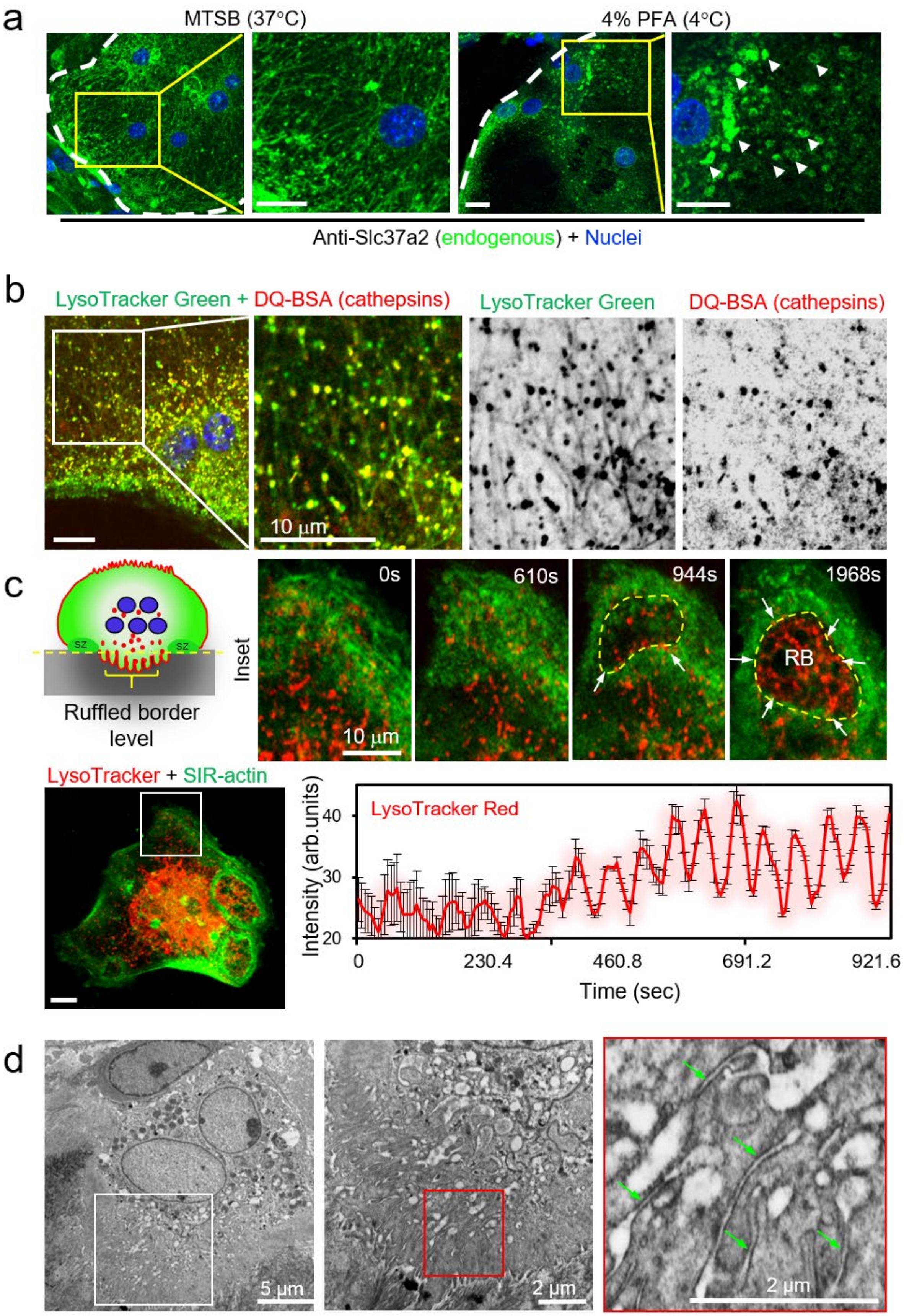

Supplementary Figure 4

Supplementary Figure 4. Tubular SLs in naïve mouse osteoclasts. Related to Fig. 4. (a) Representative immunofluorescence confocal images of endogenous Slc37a2 localization in mouse bone-marrow monocyte (BMM) derived osteoclasts fixed with microtubule stabilizing buffer (MTSB) ate 37°C or cold fixed (4°C) with 4% paraformaldehyde (PFA). White dashed lines denotes cell boundary. Arrows in magnifications highlight large Slc37a2-puncta. Bar, 10 µm, (*n* = 3). **(b)** Confocal images of a live mouse BMM-derived osteoclast culture on glass and probed with LysoTracker Green and DQ-BSA (*n* = 4). (**c**) Confocal microscopy images of live mature primary osteoclast grown on glass pulsed with LysoTracker Red and SIR-actin with enlarged time-lapse inlay and fluorescent intensity (arbitrary units, arb. units) of LysoTracker Red signal during the indicated time-course. Data are presented as mean fluorescent intensity of LysoTracker Red within F-actin rings ±SD (*n* = 3 individual F-actin rings). Source data are provided as a Source Data file. Osteoclast cartoon depicted in panels c is adapted from [1]. (**d**) Electron micrograph of a representative mouse osteoclast lining trabecular bone within the primary spongiosa of 5-day-old mice. Magnified picture illustrate tubular SL-like structures (green arrows) in the vicinity of the nascent ruffled border (*n* = 2). See also Supplementary Movie 5.

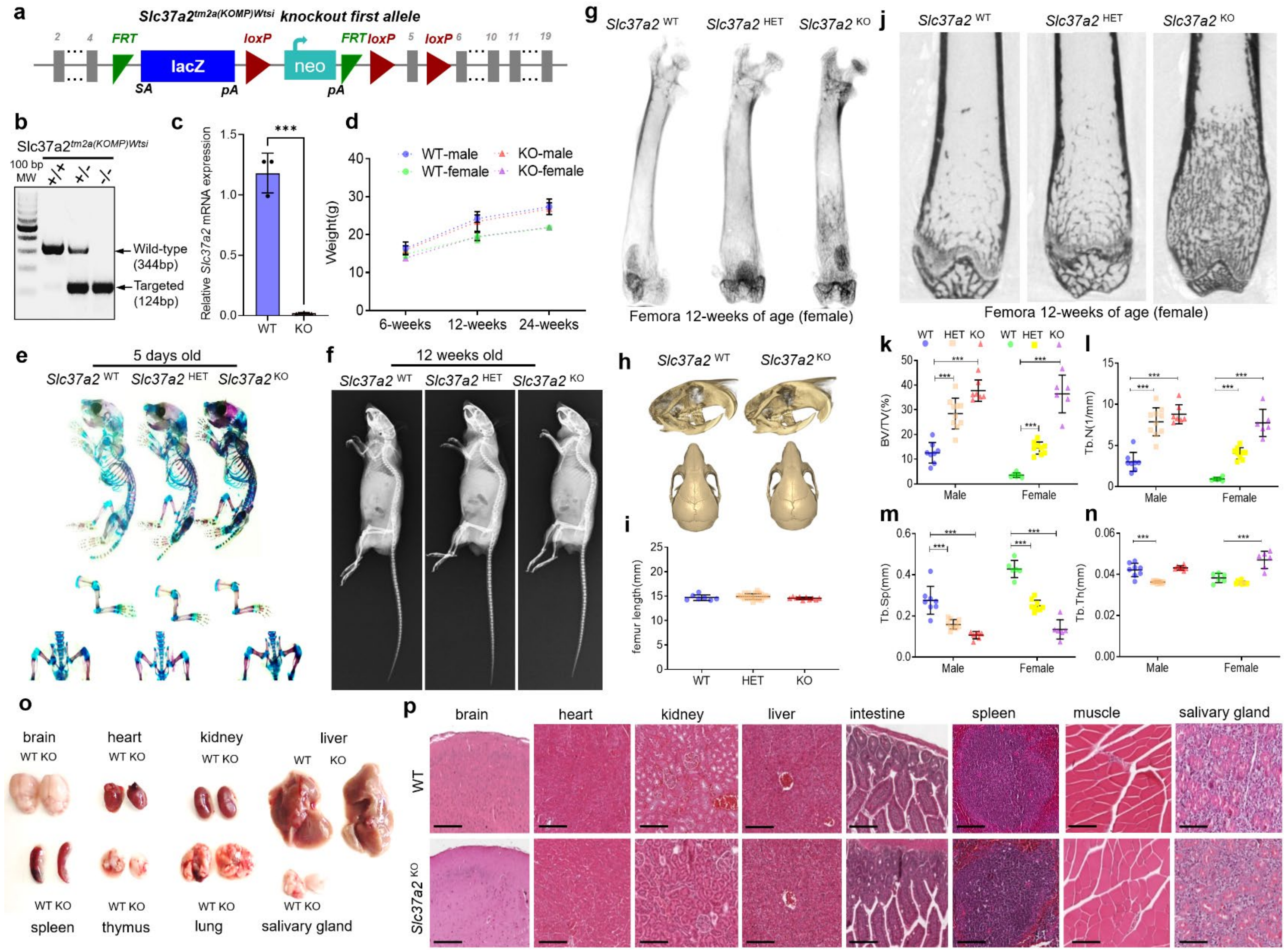

Supplementary Figure 5

Supplementary Figure 5. Generation and phenotyping of *Slc37a2*^{KO} mice. Related to Fig. 5.
(**a**) Schematic of the *Slc37a2*$^{tm2a(KOMP)Wtsi}$ knockout first allele targeting strategy. (**b**) Genotyping confirming disruption of the *Slc37a2* gene locus. WT, wildtype (+/+); HET, heterozygous (-/+); KO, homozygous (-/-). (**c**) qPCR confirmation of *Slc37a2* mRNA depletion in femurs (n = 3, P=0.0003). (**d**) Body weight of age and sex matched WT and *Slc37a2*^{KO} mice. Male mice (WT: 6-weeks n = 9, 12-weeks n = 10, 24-weeks n = 8; *Slc37a2*^{KO}: 6-weeks n = 6, 12-weeks n = 18, 24-weeks n = 8) and female mice (WT: 6-weeks n = 7, 12-weeks n = 9, 24-weeks n = 6; *Slc37a2*^{KO}: 6-weeks n = 7, 12-weeks n = 9, 24-weeks n = 5). (**e**) Five-day-old whole-mount skeletal preparations stained for Alcian blue (cartilage) and Alizarin red (bone). (**f**) Whole body mammography of 12-week old female *Slc37a2* WT, HET and KO mice. (**g-h**) Representative µCT reconstructed images of femurs (**g**), skulls (**h**) from 12-week-old female mice with genotypes indicated. (**i**) Quantification of femur length of 12-week-old-female *Slc37a2* WT (n = 7), HET (n = 22) and KO (n = 10) mice. (**j-n**) Representative sagittal µCT section (**j**) and µCT analysis (**k-n**) of distal femurs of 12-wk-old male (WT: n = 8; HET: n =10; KO: n = 7) and female (WT: n = 6; HET: n = 9; KO: n = 6) *Slc37a2* WT, HET and KO mice. (**o**) Macroscopic overview of organs from WT and *Slc37a2*^{KO} mice. (**p**) Representative H&E staining of paraffin-embedded tissue sections of WT and Slc37a2^{KO} mice. Original magnification: ×100. Bars, 200 µm. All data represent mean ± SD. *** P <0.001 by two-tailed unpaired Student's t test (**c, d**) and by two-way ANOVA with Tukey's multiple comparison test (**i, k-n**). (**k**, male WT vs HET P<0.0001; WT vs KO P<0.0001; female WT vs HET P=0.0003; WT vs KO P<0.0001; **l**, male WT vs HET P<0.0001; WT vs KO P<0.0001; female WT vs HET P<0.0001; WT vs KO P<0.0001; **m**, male WT vs HET P<0.0001; WT vs KO P<0.0001; female WT vs HET P<0.0001; WT vs KO P<0.0001; **n**, male WT vs HET P<0.0001; WT vs KO P<0.0001; female WT vs HET P<0.0001; WT vs KO P<0.0001 Source data are provided as a Source Data file.

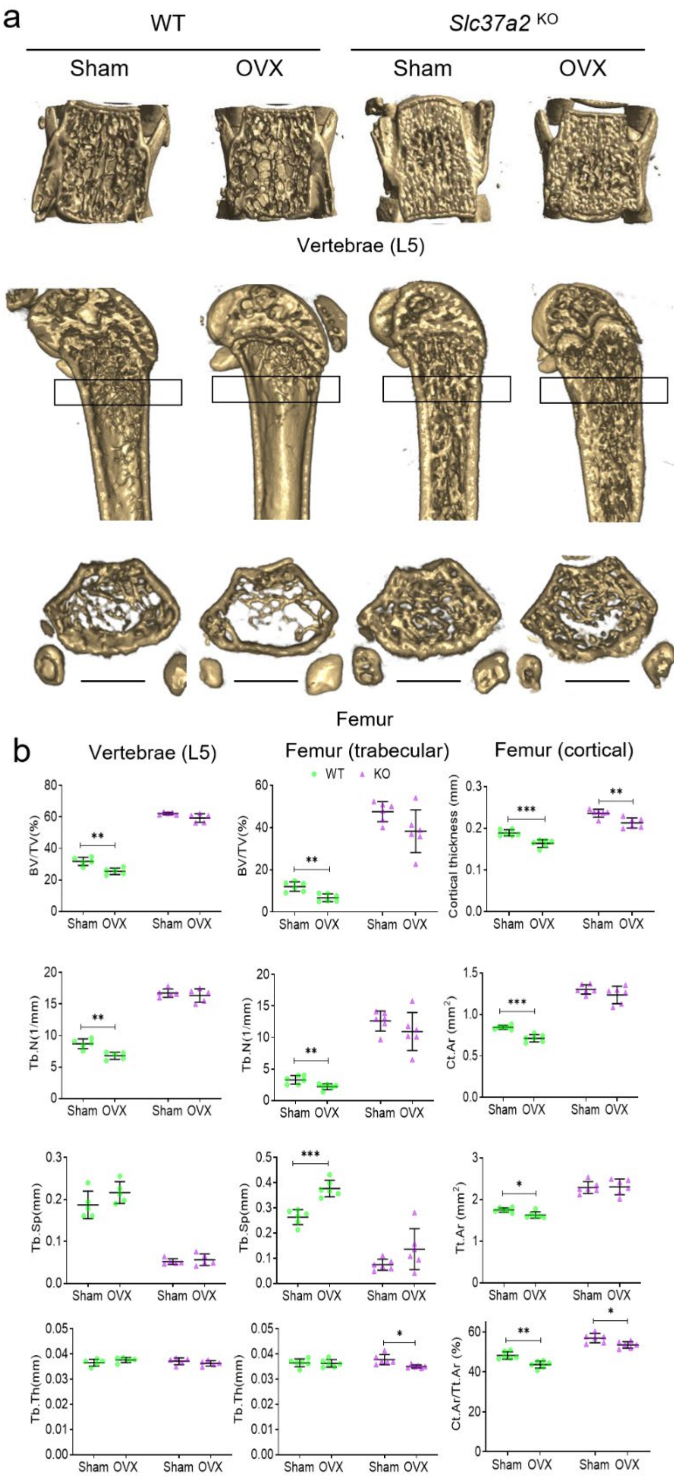

Supplementary Figure 6. *Slc37a2* deletion attenuates bone loss caused by estrogen deficiency, Related to Fig. 5. (**a**) Representative µCT images of vertebra (L5) and distal femurs and of sham operated or ovariectomized (OVX) WT and *Slc37a2*^{KO} mice. (**b**) µCT analysis of the vertebra and femurs of sham operated or OVX WT and *Slc37a2*^{KO} mice (n = 6). All data are presented as mean ± SD. *p < 0.05; **p < 0.01; ***p < 0.001; two-tailed unpaired Student's t-test. Vertebrae Bone Volume/Tissue Volume, BV/TV WT P=0.00297, Trabecular Number, Tb.N WT P=0.00257; Femur (trabecular) BV/TV WT P=0.005, Tb.N WT P=0.0098, Trabecular Separation, WT Tb.S P<0.0001, Tb.Th *Slc37a2*^{KO} P=0.01882; Femur (cortical) Cortical thickness WT P=0.00040, *Slc37a2*^{KO} P=0.00438, Cortical Area, Ct.Ar WT P=0.00023, Trabecular Area, Tt.Ar WT P=0.01252, Ct.Ar/Tt.Ar WT P=0.00133, *Slc37a2*^{KO} P=0.01738. Source data are provided as a Source Data file.

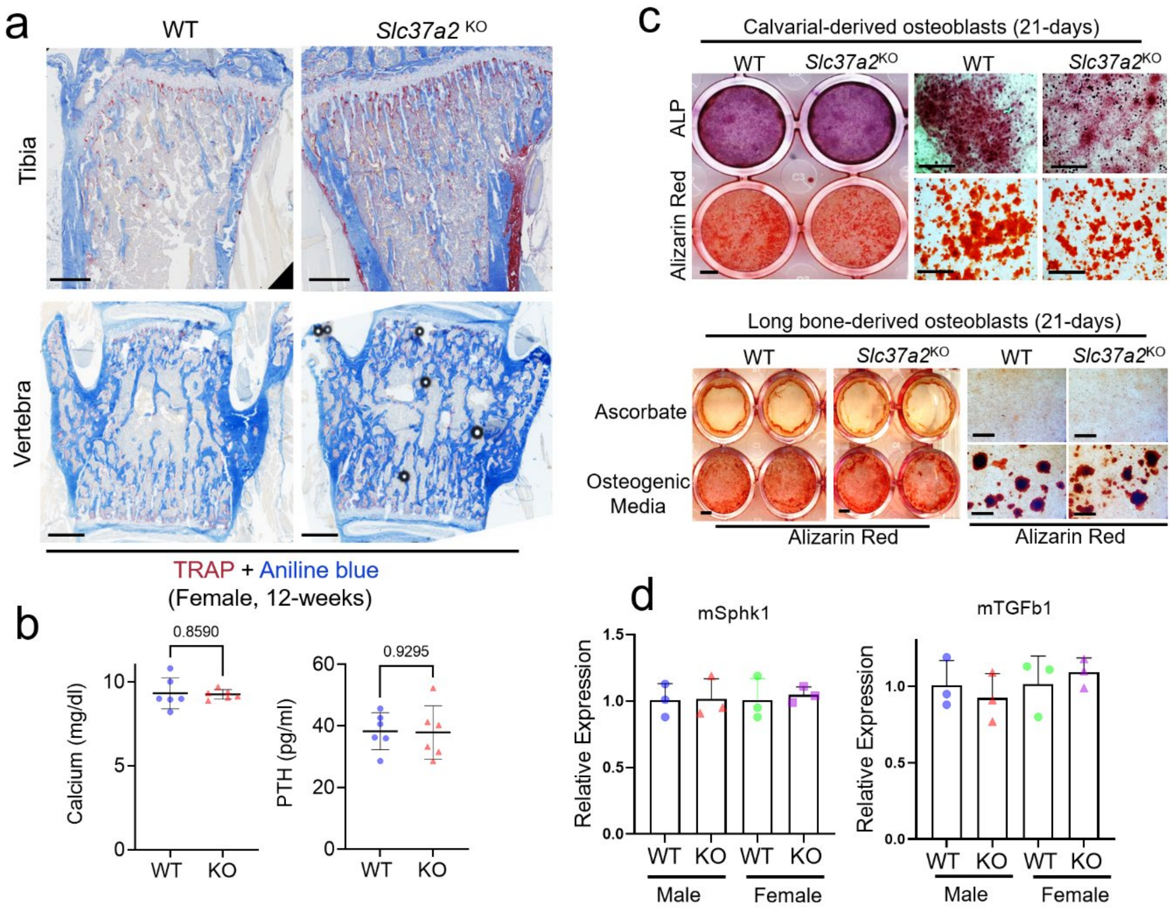

Supplementary Figure 7. (**a**) Representative histological images of Alcian blue staining of tibia and vertebra of 12-week-old female WT and *Slc37a2*^{KO} mice**.** Bars, 1 mm. (**b**) Serum calcium and parathyroid hormone (PTH) levels in 12-week-old male WT and *Slc37a2*^{KO} mice (n = 6, per genotype). Data are presented as means ±SD. (**c**) Representative alkaline phosphatase (ALP) and alizarin red staining of WT and *Slc37a2*^{KO} osteoblasts isolated from calvarial bone following culture in osteogenic media (50 µg/ml L-ascorbic acid, 2 mM β-glycerophosphate, 10^{-8} M dexamethasone) for 21-days and alizarin red staining of WT and *Slc37a2*^{KO} of mesenchymal stem cell-derived osteoblasts isolated from long bones cultured *in vitro* in the absence or presence of osteogenic media for 21 days. Bars, whole well 2 mm, magnifications 500 µm (**d**) qPCR expression of Sphk1 and TGF-β1 in mouse bone marrow monocyte-derived osteoclasts from male and female WT and *Slc37a2*^{KO} mice (n = 3, per sex/genotype). Data are presented as means ±SD. *P* values are derived by two-tailed unpaired Student's t-test. Source data are available in Source Data file.

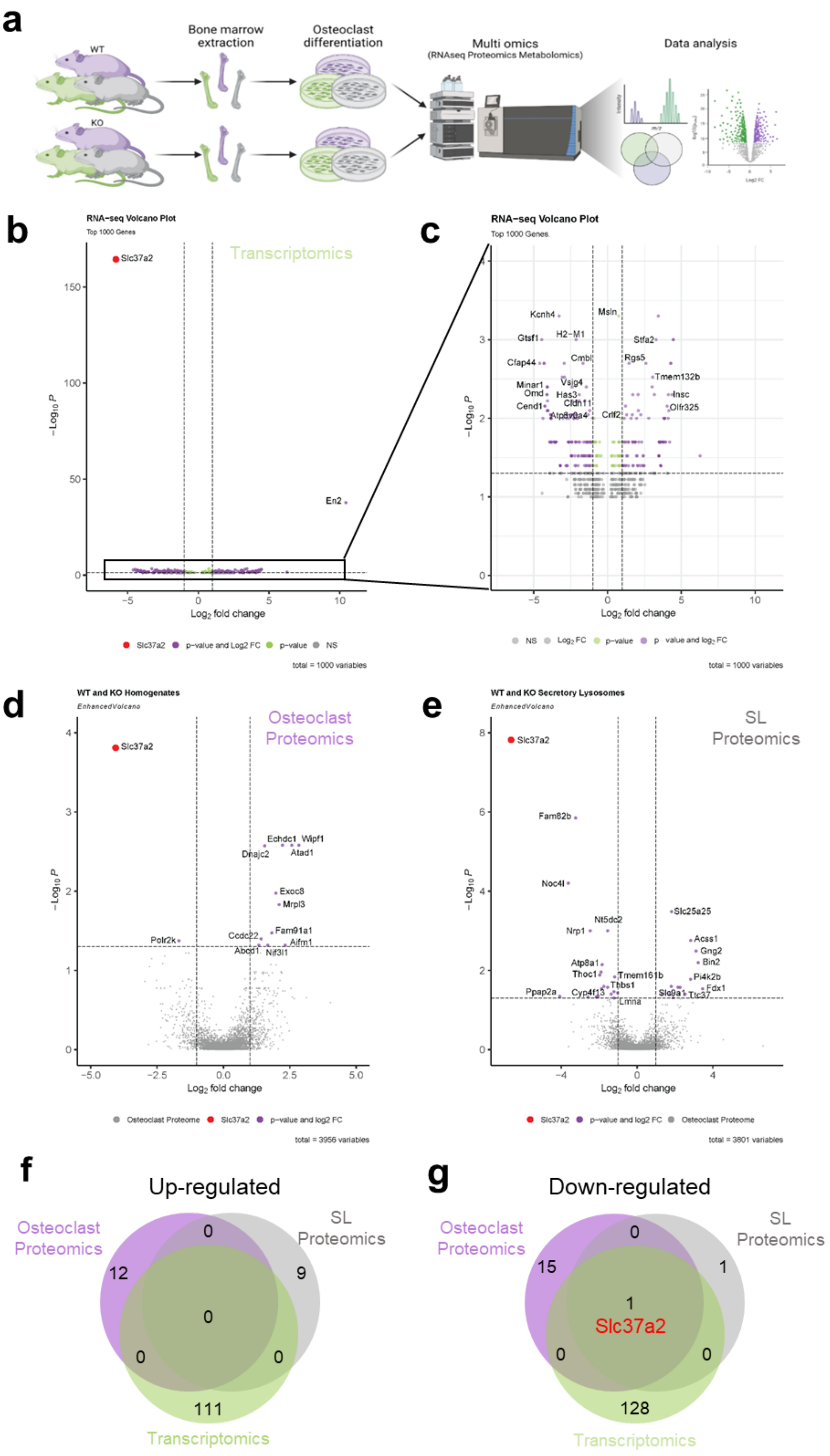

Supplementary Figure 8

Supplementary Figure 8. Multi-omic analyses of wild-type and *Slc37a2*^{KO} osteoclasts. Related to Fig. 9. (**a**) Schematic of the sample acquisition and omic tools used to compose the osteoclast proteome, transcriptome and metabolome. Created with BioRender.com. (**b**) Volcano plot summarizing the differences between the WT and *Slc37a2*^{KO} osteoclast transcriptomes (*n* = 3 biological replicates per genotype). Differentially expressed genes were defined by having an absolute number of Log_2fold-change greater than 1.5 and Benjamini-Hochberg adjusted q-value <0.05. The dashed lines represent thresholds used to identify differentially upregulated/downregulated genes. Slc37a2 is depicted in red. (**c**) Magnification of the volcano plot in (**B**) summarizing the differences between the WT and *Slc37a2*^{KO} osteoclast transcriptomes. (**d**) Volcano plot summarizing the differences between the WT and *Slc37a2*^{KO} osteoclast cellular proteomes (*n* = 3 biological replicates per genotype). Points indicate different proteins that display both magnitude fold-changes (FC, x axis) and high statistical significance (-log10 of *P* values, y axis). Dashed horizontal line shows the *P* value cut-offs (adjust *P* >0.05, ANOVA, Benjamini-Hochberg adjusted) and the two vertical dashed lines indicate down (FC -<1.5)/ up (FC>1.5) regulated proteins. (**e**) Volcano plot summarizing the differences between the WT and *Slc37a2*^{KO} osteoclast SL proteomes (*n* = 3 biological replicates per genotype). –Log10 of the adjusted *P* value (ANOVA, Benjamini-Hochberg adjusted) of the ratio between homogenate and lysosome peptide abundance versus the normalised abundance ratio Log_2FC of osteoclast whole cell homogenate and enriched secretory lysosomes. (**f**) Venn diagram summarizing the intersection between up-regulated hits (*P* >0.05, FC>1.5) from the transcriptomic and proteomic analyses; (**g**) Venn diagram summarizing the intersection between down-regulated hits (*P* >0.05, FC-<1.5) from the transcriptomic and proteomic analyses.

Supplementary References

1 Ng, P. Y., Brigitte Patricia Ribet, A. & Pavlos, N. J. Membrane trafficking in osteoclasts and implications for osteoporosis. *Biochemical Society transactions*, doi:10.1042/BST20180445 (2019).

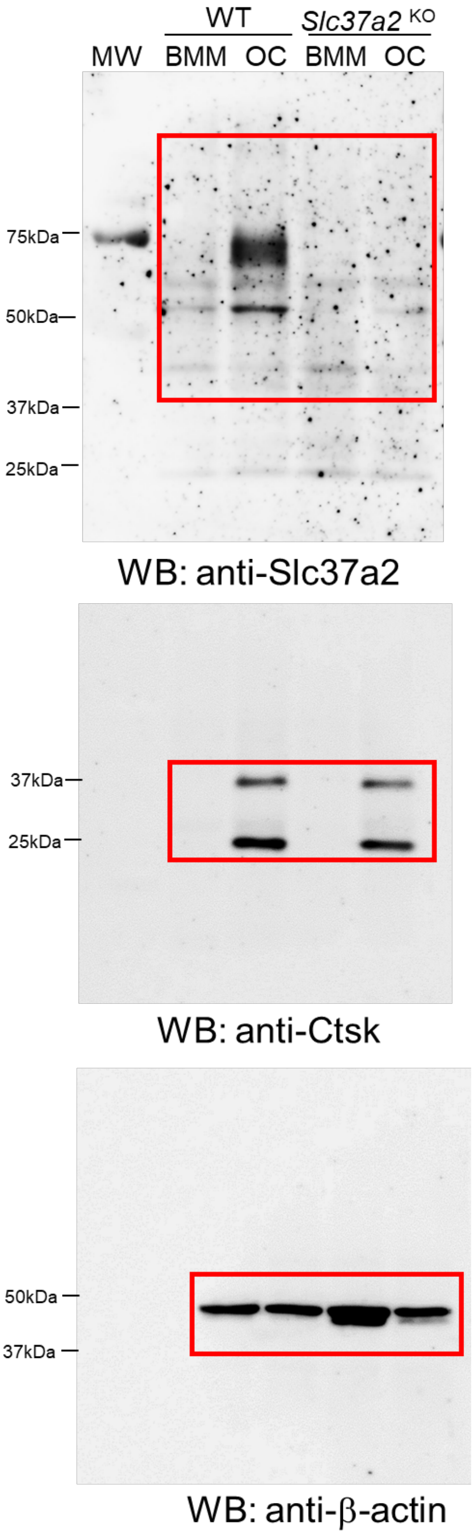

Supplementary Fig. 1a

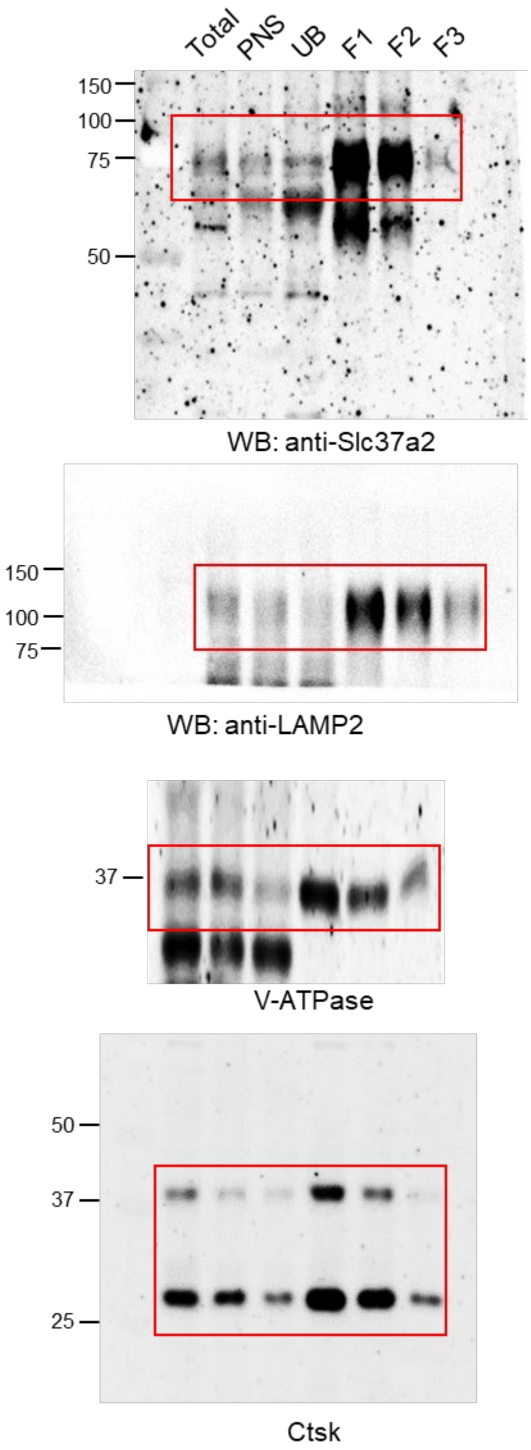

Supplementary Fig. 2a

Uncropped blots for Supplementary Figure 1a and Supplementary Figure 2a
