# Supplementary material for: Sugar transporter Slc37a2 regulates bone metabolism in mice via a tubular lysosomal network in osteoclasts
Source: Nat Commun. 2023 Feb 21;14:906. doi: 10.1038/s41467-023-36484-2 (PMC9945426; doi:10.1038/s41467-023-36484-2)
Supplement: Supplementary file 3 — Description of Additional Supplementary Files [file 41467_2023_36484_MOESM3_ESM.pdf]

Description of Additional Supplementary Files

Supplementary Data 1: The osteoclast secretory lysosome (SL) proteome. Related to Fig. 1.

Supplementary Movie 1: Slc37a2 localizes to a dynamic network of acidified tubular lysosomes in osteoclasts which house cathepsin K, Related to Fig. 3. Representative time-lapse video of emGFP-Slc37a2 in osteoclasts with indicated marker proteins cortactin-dtTomato (a), LysoTracker (b), mCherry-VAMP7 (c) and Cathepsin K Magic Red (MR) (D). Yellow circles denote nuclei. Frames were taken at 3.9 s intervals.

Supplementary Movie 2: emGFP-Slc37a2+ tubular SLs are morphologically and dynamically distinct from endosomes, Related to Fig.3 and Supplementary Fig. 2b. Representative time-lapse video of emGFP-Slc37a2 and early endosome marker mCherry-Rab5 in osteoclasts. Frames were taken at 3.9 s intervals.

Supplementary Movie 3: emGFP-Slc37a2+ tubular SLs are acidified and fuse with the osteoclast plasma membrane, Related to Fig. 3. Representative time-lapse video of an emGFP-Slc37a2 expressing osteoclast cultured on glass labeled with indicated markers. Yellow dashed line denotes plasma membrane and red arrows highlight fusion events in magnified view. Frames were taken at 3.9 s intervals.

Supplementary Movie 4: Slc37a2+ tubular SLs orientate towards the ruffled border and transiently fuse with the bone-lining plasma membrane, Related to Fig. 4. Representative time-lapse video of emGFP-Slc37a2 in an osteoclast cultured on bone co-labeled with LysoTracker and viewed at the level of the ruffled border (yellow dashed outline). Arrows on magnified images highlight a fusion event.

Supplementary Movie 5: Osteoclasts possess dynamic tubular SLs which store cathepsins and form a network within F-actin rings, Related to Fig. 4 and Supplementary Fig. 4b-c. Representative time-lapse video of mouse BMM-derived osteoclasts cultured on either glass (a) or bone (b) and labeled with the indicated vital markers. Arrows denote ruffled border (yellow) and F-actin ring/sealing zone (green), respectively.

Supplementary Movie 6: Invertase-induced sucrosome resolution and tubulation is impaired in Slc37a2KO osteoclasts, Related to Fig. 9. Representative time-lapse video of LysoTracker-labeled sucrosomes in WT and Slc37a2KO osteoclasts 1 h after the addition of invertase to induced sucrosome resolution and membrane tubulation. Arrows highlight tubulation events. Frames were taken at 3.9 s intervals for 10 min, playback at 11 fps.
