# Supplementary material for: Sugar transporter Slc37a2 regulates bone metabolism in mice via a tubular lysosomal network in osteoclasts
Source: Nat Commun. 2023 Feb 21;14:906. doi: 10.1038/s41467-023-36484-2 (PMC9945426; doi:10.1038/s41467-023-36484-2)
Supplement: Supplementary file 11 — Reporting Summary [file 41467_2023_36484_MOESM11_ESM.pdf]

nature portfolio

Corresponding author(s): Nathan John Pavlos

Last updated by author(s): Jan 3, 2023

Reporting Summary

Nature Portfolio wishes to improve the reproducibility of the work that we publish. This form provides structure for consistency and transparency in reporting. For further information on Nature Portfolio policies, see our Editorial Policies and the Editorial Policy Checklist.

Statistics

For all statistical analyses, confirm that the following items are present in the figure legend, table legend, main text, or Methods section.

n/a	Confirmed	
[ ]	[x]	The exact sample size (*n*) for each experimental group/condition, given as a discrete number and unit of measurement
[ ]	[x]	A statement on whether measurements were taken from distinct samples or whether the same sample was measured repeatedly
[ ]	[x]	The statistical test(s) used AND whether they are one- or two-sided<br>*Only common tests should be described solely by name; describe more complex techniques in the Methods section.*
[x]	[ ]	A description of all covariates tested
[x]	[ ]	A description of any assumptions or corrections, such as tests of normality and adjustment for multiple comparisons
[ ]	[x]	A full description of the statistical parameters including central tendency (e.g. means) or other basic estimates (e.g. regression coefficient) AND variation (e.g. standard deviation) or associated estimates of uncertainty (e.g. confidence intervals)
[ ]	[x]	For null hypothesis testing, the test statistic (e.g. *F*, *t*, *r*) with confidence intervals, effect sizes, degrees of freedom and *P* value noted<br>*Give P values as exact values whenever suitable.*
[x]	[ ]	For Bayesian analysis, information on the choice of priors and Markov chain Monte Carlo settings
[x]	[ ]	For hierarchical and complex designs, identification of the appropriate level for tests and full reporting of outcomes
[ ]	[x]	Estimates of effect sizes (e.g. Cohen's *d*, Pearson's *r*), indicating how they were calculated

Our web collection on statistics for biologists contains articles on many of the points above.

Software and code

Policy information about availability of computer code

Data collection	Proteome Discoverer™ 2.3 software, NIKON A1R in a Ti-E inverted motorized confocal microscope, microCT systems (Skyscan1176, Carl Zeiss X-ray Microscope Versa 520), Agilent MassHunter software package (B.05.00), NovaSeq Control Software (NCS) v1.7.0 and Real Time Analysis (RTA) v3.4.4.
Data analysis	Proteome Discoverer™ 2.3 software, BioQuant Osteo version 13.2.6, GraphPad Prism 9.0.2, VErsatile Gene-based Association Study 2 (VEGAS2) software package, NIS Elements AR software (Nikon) version 5.30.02 (Build 1545), ImageJ (Fiji) 1.53c software, Nikon digital sight DS-5MC and NIS BR Elements 3.2 software, Skyscan CTvox software version3.0.0r1114, NRecon version 1.6.10.4 (64bit), Bruker CTAn (CT-analyser) version 1.14.4.1, XMReconstructor v10.7.36.79.13921, TXM3DViewer workstation 1.2, AMDIS software (v 2.73), edgeR (version 3.30.3) in R4.0.0.

For manuscripts utilizing custom algorithms or software that are central to the research but not yet described in published literature, software must be made available to editors and reviewers. We strongly encourage code deposition in a community repository (e.g. GitHub). See the Nature Portfolio guidelines for submitting code & software for further information.

Data

Policy information about availability of data

All manuscripts must include a data availability statement. This statement should provide the following information, where applicable:

- Accession codes, unique identifiers, or web links for publicly available datasets
- A description of any restrictions on data availability
- For clinical datasets or third party data, please ensure that the statement adheres to our policy

The proteomic data that support the findings of this study have been deposited to the ProteomeXchange Consortium via the PRIDE partner repository under accession numbers PXD037006, PXD037813 and PXD037910. The RNAseq data produced in this study were deposited to the public data base (GSE219216: https://www.ncbi.nlm.nih.gov/geo/query/acc.cgi?acc=GSE219216). All data supporting the findings of this study are available within the article and its Supplementary Information files. Source Data are provided with this paper.

Human research participants

Policy information about studies involving human research participants and Sex and Gender in Research.

Reporting on sex and gender	This study did not involve human research participants.
Population characteristics	N/A
Recruitment	N/A
Ethics oversight	N/A

Note that full information on the approval of the study protocol must also be provided in the manuscript.

Field-specific reporting

Please select the one below that is the best fit for your research. If you are not sure, read the appropriate sections before making your selection.

- [x] Life sciences
- [ ] Behavioural & social sciences
- [ ] Ecological, evolutionary & environmental sciences

For a reference copy of the document with all sections, see nature.com/documents/nr-reporting-summary-flat.pdf

Life sciences study design

All studies must disclose on these points even when the disclosure is negative.

Sample size	No statistical method was used to predetermine the sample size. Sample size was determined based on the numbers required to achieve statistical significance using non-parametric studies and our prior experience from similar published studies (Chan, A.S. et al., MBoC, 27, 1367-1382, 2016). For in vitro experiments, littermates were used and experiments repeated with biological and technical replicates to ensure reproducibility.
Data exclusions	No data were excluded from the analyses.
Replication	Results are representative from at least two or three biological replicates per experiment. All attempts at replication were successful. The number of biological replicates (n) is reported for each experiment.
Randomization	Allocation of mice was random in all in vivo experiments, taken from littermates. For in vitro analyses, cells were randomly allocated to experimental groups.
Blinding	Investigators were blinded to group allocation during data collection and/or analysis of all in vivo experiments. In vitro analyses of osteoclast resorption pit numbers/size/trenches was also performed by an investigator blinded to the culture genotypes. Blinding was also performed for microscopic assessment of osteoclast ruffled border morphology and lysosomal quantitation. Blinding was not relevant for immunoblot and other immunofluorescence analyses performed exclusively on wildtype animals.

Reporting for specific materials, systems and methods

We require information from authors about some types of materials, experimental systems and methods used in many studies. Here, indicate whether each material, system or method listed is relevant to your study. If you are not sure if a list item applies to your research, read the appropriate section before selecting a response.

Materials & experimental systems

n/a	Involved in the study
	☒ Antibodies
	☒ Eukaryotic cell lines
☒	☐ Palaeontology and archaeology
	☒ Animals and other organisms
☒	☐ Clinical data
☒	☐ Dual use research of concern

Methods

n/a	Involved in the study
☒	☐ ChIP-seq
☒	☐ Flow cytometry
☒	☐ MRI-based neuroimaging

Antibodies

Antibodies used

A Slc37a2-specific polyclonal antibody was generated by immunizing rabbits with the peptide 'CTPPRHHDDPEKEQ,' corresponding to the cytoplasmic facing intracellular loop of mouse Slc37a2. Antisera were affinity-purified against the corresponding immunization-peptide. Slc37a2 peptide antibodies were produced using the PolyExpressTM service by GenScript (New Jersey, USA).Antibodies were used at 1:100 dilution for immunostaining and 1:1000 dilution for immunoblotting experiments.

Other primary antibodies used (name, clone, supplier, catalogue number, dilution):
Mouse monoclonal anti-Actin JLA20, deposited by Lin, J.J.-C DSHB JLA20 IB 1:2000
Rabbit monoclonal anti-Akt (pan) C67E7 Cell Signaling Technology 4691 IB 1:1000
Rabbit monoclonal anti-Phospho-Akt (Ser473) D9E XP®, Cell Signaling 4060 IB 1:1000
Mouse monoclonal anti-ARL8A/B H-8 Santa Cruz sc-398635 IF 1:200
Mouse monoclonal anti-Cathepsin K 182-12G5 Millipore MAB3324 IF 1:300
Mouse monoclonal anti-Cathepsin K E-7 Santa Cruz sc-48353 IB: 1:250
Rabbit polyclonal anti-Cathepsin B Atlas Antibodies HPA018156 IB 1:250
Mouse monoclonal anti-CD63/LAMP3 H5C6 DSHB H5C6 IF 1:100
Rabbit polyclonal anti-Collagen I Abcam ab34710 IF 1:300
Rabbit polyclonal anti t-ERK1/2 Promega V1141 IB 1:1000
Mouse monoclonal anti-p-ERK1/2 E-4 Santa Cruz sc-7383 IB 1:500
Mouse monoclonal anti-GM130 35/GM130 BD Transduction Laboratories 610822 IF 1:100
Rabbit polyclonal anti- IκBα C-21 Santa Cruz sc-371 IB 1:1000
Rat monoclonal anti-LAMP-2 ABL-93, deposited by August, J.T DSHB ABL-93 IB 1:1000
Rat monoclonal anti-LAMP-2 GL2A7, deposited by Granger, B. L DSHB GL2A7 IF 1:100
Mouse monoclonal anti-NFATc1 7A6, deposited by Crabtree, G.R DSHB 7A6 IB 1:1000
Rabbit polyclonal anti-MMP13 ab39012 Abcam ab39012 IF 1:300
Mouse monoclonal anti-PDI 1D3 ENZO Life Sciences ADI-SPA-891-F IF 1:200
Rabbit polyclonal anti-Rab1b Santa Cruz sc-599 IB 1:5000
Mouse monoclonal anti-Rab5 621.3 Synaptic Systems 108 011 IB 1:1000
Rabbit monoclonal anti-Rab7 D95F2 Cell Signaling Technology 9367 IF 1:100
Mouse monoclonal anti-Rab7 E9O7E Cell Signaling Technology 95746 IB 1:2000
Mouse monoclonal anti-Rab38 A-8 Santa Cruz sc-390176 IB 1:200
Rabbit monoclonal anti-RUNX2 D1L7F Cell Signaling Technology 12556 IF 1:200
Rabbit polyclonal anti-Syntaxin 16 Synaptic Systems 110 163 IB 1:300
Mouse monoclonal anti-c-Src GD11 Millipore 05-184 IB 1:1000
Mouse monoclonal V-ATPase D1 34-Z Santa Cruz sc-81887 IB 1:1000
Rabbit polyclonal anti-VDAC3 H-40 Santa Cruz sc-292328 IB 1:500
Rabbit polyclonal anti-VGluT-1 Synaptic Systems 135 303 IB: 1:1000
Mouse monoclonal anti-Vps35 B-5 Santa Cruz sc-374372 IF: 1:300

Secondary Antibodies used:
Goat anti-mouse IgG (Fab specific)-Peroxidase antibody Sigma-Aldrich A9917 IB 1:5000
Goat anti-rabbit IgG (whole molecule)-Peroxidase antibody Sigma-Aldrich A0545 IB 1:5000
Alexa Fluor 488 goat anti-mouse IgG (H+L), highly cross-adsorbed Thermo Fisher Scientific A-11029 IF: 1:500
Alexa Fluor 568 goat anti-mouse IgG (H+L), highly cross-adsorbed Thermo Fisher Scientific A-11031 IF: 1:500
Alexa Fluor 647 goat anti-mouse IgG (H+L), highly cross-adsorbed Thermo Fisher Scientific A-21236 IF: 1:500
Alexa Fluor 488 goat anti-rabbit IgG (H+L), highly cross-adsorbed Thermo Fisher Scientific A-11034 IF: 1:500
Alexa Fluor 568 goat anti-rabbit IgG (H+L), highly cross-adsorbed Thermo Fisher Scientific A-11036 IF 1:500
Alexa Fluor 647 goat anti-rabbit IgG (H+L), highly cross-adsorbed Thermo Fisher Scientific A-21245 IF 1:500
Alexa Fluor 555 donkey anti-rat, IgG (H+L), highly cross-adsorbed Thermo Fisher Scientific A-48270 IF 1:500

Validation

Rabbit polyclonal anti-mouse Slc37a2 primary antibody was validated by GenScript PolyExpress service by ELISA and specificity was confirmed against pre-immune serum, control peptide and immunoblotting against Slc37a2-deficient mouse osteoclasts.
All other antibodies were commercially sourced with validation statements available on the following manufactures' websites:
Actin https://dshb.biology.uiowa.edu/JLA20; AKT (pan) https://www.cellsignal.com/products/primary-antibodies/akt-pan-c67e7-rabbit-mab/4691; Phospho-Akt (Ser473) https://www.cellsignal.com/products/primary-antibodies/phospho-akt-ser473-d9e-xp-

rabbit-mab/4060; ARL8A/B https://www.scbt.com/p/arl8a-b-antibody-h-8; Cathepsin K https://www.merckmillipore.com/INTERSHOP/web/WFS/Merck-AT-Site/de_DE/-/EUR/ShowDocument-File?ProductSKU=MM_NF-MAB3324&DocumentId=null&DocumentUID=16910422&DocumentType=COA&Language=EN&Country=US&ProductBatchNo=2583409&Origin=PDP; Cathepsin K (E-7) https://www.scbt.com/p/cathepsin-k-antibody-e-7; Cathepsin B https://www.sigmaaldrich.com/AU/en/product/sigma/hpa018156; CD63/LAMP3 https://dshb.biology.uiowa.edu/H5C6; Collagen 1 https://www.abcam.com/collagen-i-antibody-ab34710.html; t-ERK 1/2 chrome-extension://efaidnbmnnnibpcajpcglclefindmkaj/https://www.promega.com/-/media/files/resources/protocols/technical-bulletins/0/anti-active-mapk-jnk-and-p38-polyclonal-antibodies-protocol.pdf?rev=2646a21713c24e258dbc96066456a6c2&sc_lang=en; p-ERK (E-4) https://www.scbt.com/p/p-erk-antibody-e-4; GM130 https://www.bdbiosciences.com/en-au/products/reagents/microscopy-imaging-reagents/immunofluorescence-reagents/purified-mouse-anti-gm130.610822 ; IkappaBalpha-https://www.scbt.com/p/ikappab-alpha-antibody-c-21; LAMP2 (ABL-93) https://dshb.biology.uiowa.edu/ABL-93; LAMP2 (GL2A7) https://dshb.biology.uiowa.edu/GL2A7; NFATc1 https://dshb.biology.uiowa.edu/7A6; MMP13 https://www.abcam.com/mmp13-antibody-ab39012.html; PDI https://www.enzolifesciences.com/ADI-SPA-891/pdi-monoclonal-antibody-1d3/; Rab1 https://www.scbt.com/p/rab-1b-antibody-g-20; Rab5 https://sysy.com/product/108011; Rab7 (D95F2) https://www.cellsignal.com/products/primary-antibodies/rab7-d95f2-xp-rabbit-mab/9367; Rab7 (E9O7E) https://www.cellsignal.com/products/primary-antibodies/rab7-e9o7e-mouse-mab/95746; Rab38 https://www.scbt.com/p/rab-38-antibody-a-8?requestFrom=search; RUNX2 https://www.cellsignal.com/products/primary-antibodies/runx2-d1l7f-rabbit-mab/12556; syntaxin 16- https://sysy.com/product/110163#list; c-Src https://www.merckmillipore.com/AU/en/product/Anti-Src-Antibody-clone-GD11,MM_NF-05-184; V-ATPase D1 https://www.scbt.com/p/v-atpase-d1-antibody-34-z?requestFrom=search; VDAC3 chrome-extension://efaidnbmnnnibpcajpcglclefindmkaj/https://datasheets.scbt.com/sc-292328.pdf; VGluT-1-https://sysy.com/product/135303; Vps35 https://www.scbt.com/p/vps35-antibody-b-5; Anti-mouse peroxidase https://www.sigmaaldrich.com/AU/en/product/sigma/a9917; Anti-rabbit peroxidase ;https://www.sigmaaldrich.com/AU/en/product/sigma/a0545; Alexa Fluor 488 goat anti-mouse https://www.thermofisher.com/antibody/product/A-11029.html; Alexa Fluor 568 goat anti-mouse https://www.thermofisher.com/antibody/product/A-11031.html; Alexa Fluor 647 goat anti-mouse https://www.thermofisher.com/antibody/product/A-21236.html; Alexa Fluor 488 goat anti-rabbit https://www.thermofisher.com/antibody/product/A-11034.html; Alexa Fluor 568 goat anti-rabbit https://www.thermofisher.com/antibody/product/A-11036.html; Alexa Fluor 647 goat anti-rabbit https://www.thermofisher.com/antibody/product/Goat-anti-Rabbit-IgG-H-L-Highly-Cross-Adsorbed-Secondary-Antibody-Polyclonal/A-21245; Alexa Fluor 555 donkey anti-rat https://www.thermofisher.com/antibody/product/Donkey-anti-Rat-IgG-H-L-Highly-Cross-Adsorbed-Secondary-Antibody-Polyclonal/A48270

Eukaryotic cell lines

Policy information about cell lines and Sex and Gender in Research

Cell line source(s)	HEK293 and BHK cell lines were acquired commercially from the ATCC. HEK293FT cells (R70007) were acquired commercially from Thermo Fisher. The JM8A3.N1 cell line (Clone ID EPD0641_4_E06) were obtained from the KOMP consortium and provided by the UC Davis KOMP Repository.
Authentication	Morphological shape of cell lines and proliferation rates were regularly monitored by microscopy.
Mycoplasma contamination	All cell lines tested negative for mycoplasma contamination.
Commonly misidentified lines (See ICLAC register)	No commonly misidentified cell lines were used.

Animals and other research organisms

Policy information about studies involving animals; ARRIVE guidelines recommended for reporting animal research, and Sex and Gender in Research

Laboratory animals	Mus musculus, C57BL/6N-SLC37A2tm2a(KOMP)Wtsi, male and female mice 6-28weeks of age. All mice were maintained on the C57BL/6N background under specific-pathogen free conditions at the Animal Resources Centre (ARC), Murdoch, Western Australia (W.A). Mice were housed in standard cages (45×29×12cm) and maintained in a temperature 22±1°C and humidity-controlled room (40–65%) on a 12 h light cycle with ad libitum access to water and a standard laboratory chow diet (SF00-100, Specialty Feeds, Glen Forrest, W.A.).
Wild animals	The study did not involve wild animals.
Reporting on sex	Sex specific data is provided for all in vivo and in vitro studies. Sex was considered in the study design for ovariectomy studies where experiments were performed exclusively on female mice.
Field-collected samples	The study did not involve samples collected from the field.
Ethics oversight	All animal procedures were approved by the Animal Ethics Committee of The University of Western Australia (Approval No. RA13/100/1475).

Note that full information on the approval of the study protocol must also be provided in the manuscript.
